# Age under 20 years, pre‐operative participation in pivoting sports, and steep posterior tibial slope of more than 12° are risk factors for graft failure after double‐bundle anterior cruciate ligament reconstruction

**DOI:** 10.1002/jeo2.70102

**Published:** 2024-12-03

**Authors:** Kazumi Goto, Eisaburo Honda, Hiroshi Iwaso, Shin Sameshima, Miyu Inagawa, Yutaro Ishida, Koji Matsuo, Ryota Kuzuhara, Takaki Sanada

**Affiliations:** ^1^ Department of Sports Orthopaedic Surgery Kanto Rosai Hospital Kawasaki Kanagawa Japan

**Keywords:** anterior cruciate ligament reconstruction, double‐bundle technique, graft failure, posterior tibial slope

## Abstract

**Purpose:**

Younger age and steep posterior tibial slope (PTS) have been reported as risk factors for graft failure after anterior cruciate ligament reconstruction (ACLR). Few studies have evaluated these risk factors simultaneously in a large cohort of patients undergoing double‐bundle ACLR (DB‐ACLR). Therefore, this retrospective study aimed to simultaneously investigate known risk factors such as PTS and age in DB‐ACLR, determine their thresholds and calculate odds ratios (ORs).

**Methods:**

We investigated 482 knees that underwent DB‐ACLR with a follow‐up period of at least 2 years. Receiver operating characteristic analysis determined cut‐off values for age and PTS for graft failure. Subsequently, logistic regression analysis was conducted to evaluate the effects of age, sex, height, weight, laterality, surgical waiting period, pre‐operative sport type and level, meniscal injury, hyperextension, general joint laxity and PTS on graft failure.

**Results:**

Graft failure was observed in 33 out of 482 knees (6.8%). Notably, the graft failure group was significantly younger (18.0 ± 5.0 years [standard deviation] vs. 30.4 ± 13.1 years, *p* < 0.01) and had a steeper PTS (11.9 ± 2.3° [standard deviation] vs. 9.6 ± 2.9°, *p* < 0.01) than the group with no graft failure. The cut‐off values were 20.0 years for age (specificity, 64.6%; sensitivity, 87.9% and area under the curve, 0.808) and 12.0° for PTS (specificity, 70.9%; sensitivity, 69.7% and area under the curve, 0.734). Logistic regression analysis identified an age of <20 years (OR = 10.1; *p* < 0.01), PTS of ≥12° (OR = 5.6; *p* < 0.01) and pre‐operative participation in pivoting sports (OR = 6.0; *p* < 0.01) as significant risk factors for graft failure.

**Conclusion:**

We identified an age of <20 years, PTS of ≥12° and pre‐operative participation in pivoting sports as significant risk factors for graft failure after DB‐ACLR.

**Level of Evidence:**

Level III.

**Abbreviations**
ACL: anterior cruciate ligament
AP: antero‐posterior
AUC: area under the curve
BTB: bone‐patellar tendon‐bone
CI: confidence interval
DB‐ACLR: double‐bundle anterior cruciate ligament reconstruction
GJL: general joint laxity
ICC: intraclass correlation coefficient
LM: lateral meniscus
LPTS: lateral posterior tibial slope
LTP: lateral tibial plateau
MM: medial meniscus
MPTS: medial posterior tibial slope
MRI: magnetic resonance imaging
MTP: medial tibial plateau
OR: odds ratio
PTS: posterior tibial slope
ROC: receiver operating characteristic

## INTRODUCTION

Double‐bundle anterior cruciate ligament reconstruction (DB‐ACLR) replicates the biomechanical knee [24, 28]; however, the re‐rupture rate remains similar to that of single‐bundle ACLR [22]. The reason for the double‐bundle technique's inability to achieve a higher success rate than the single‐bundle technique remains unclear [7], despite its better replication of the native knee [34]. Furthermore, while some studies have examined residual pivot‐shift after DB‐ACLR [16, 31] and others have investigated graft laxity [35, 37], detailed evaluations of the age and posterior tibial slope (PTS) thresholds are limited.

Previous reports have indicated different age thresholds for increased risk of graft failure, with some studies identifying individuals aged under 18 years [33] as having an increased risk and others indicating thresholds of 20 [8], 21 [21] and 22 years [38]. However, there is no consensus on the exact age threshold to define ‘younger’ individuals at higher risk of graft failure after ACLR [26]. Similarly, PTS thresholds vary from 10° to 17° across different studies [5, 17, 23]. These studies used different measurement methods—with some using X‐rays and others using magnetic resonance imaging (MRI)—and measurement sites (varying between the medial tibial plateau [MTP] and lateral tibial plateau [LTP]) [20]. Research on these risk factors often verifies individual factors using propensity score matching [5, 23] or comprehensively investigates them through systematic reviews [20, 38]. However, few studies have examined multiple risk factors within the same cohort.

This study aimed to simultaneously investigate known risk factors such as PTS and age in DB‐ACLR, determine their thresholds and calculate odds ratios (ORs). We hypothesised that the risk factors for graft failure in DB‐ACLR include a younger age, a steep PTS angle, meniscal injury, general joint laxity (GJL) and knee hyperextension. Understanding these risk factors can help predict which individuals are likely to have a lower risk of graft failure after DB‐ACLR.

## MATERIALS AND METHODS

**Figure 1:**
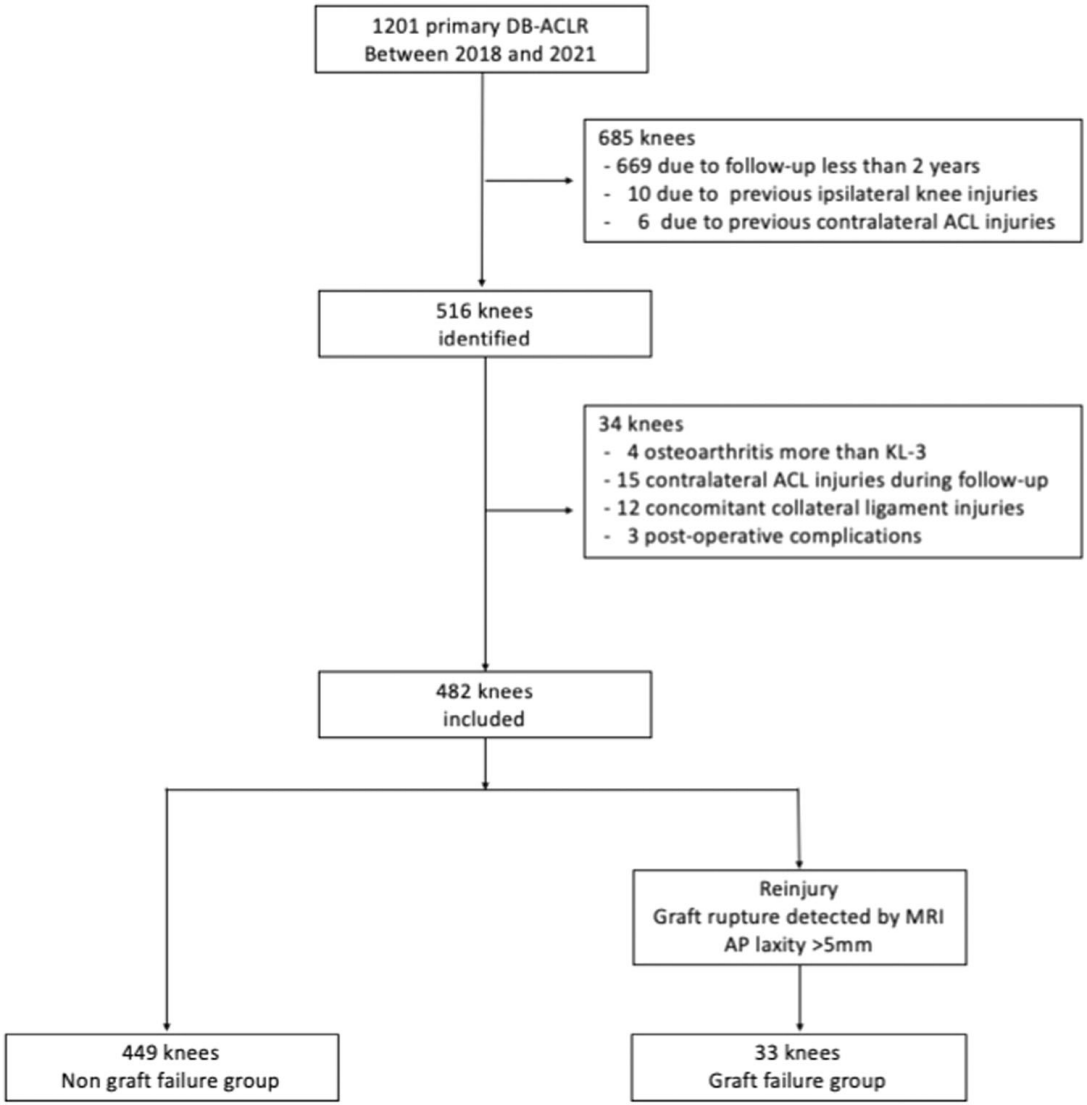
Flowchart illustrating the criteria for inclusion in and exclusion from the study. ACL, anterior cruciate ligament; AP, anteroposterior; DB‐ACLR, double‐bundle anterior cruciate ligament reconstruction; KL, Kellgren–Lawrence; MRI, magnetic resonance imaging.

This retrospective cohort study was approved by the institutional review board (IRB: Kanto Rosai Hospital 2024‐11) and included 1201 patients diagnosed with primary ACL injuries who underwent DB‐ACLR with hamstring autografts at our institution between 2018 and 2021. Only patients with a follow‐up period of at least 2 years were included. The exclusion criteria were as follows: previous ipsilateral knee injuries, previous contralateral ACL injuries, osteoarthritis greater than Kellgren–Lawrence Grade 3, contralateral ACL injuries during follow‐up, concomitant collateral ligament injuries (Grade 2 or 3), and post‐operative complications such as surgical site infection and severe arthrofibrosis (Figure 1). Ultimately, 482 patients were included in this study and we collected the following data from their medical records: age, sex, height, weight, laterality, surgical waiting period, pre‐operative sports type, pre‐operative Tegner Activity Scale, presence of meniscal injury, knee hyperextension of ≥10°, GJL and PTS (Table 1). The pre‐operative sports type was classified as pivoting or non‐pivoting, with pivoting sports defined as sports frequently involving jumping, cutting and pivoting movements (e.g., basketball, soccer, futsal, American football, rugby, tennis, lacrosse, handball and badminton), based on previous research [2, 19].

**Table 1:** Patient demographics.

	Non‐graft failure group (*n* = 449)	Graft failure group (*n* = 33)	*p*
Age (years)	30.4 ± 13.1	18.0 ± 5.0	<0.01
Sex (female/male)	245/204	21/12	0.37
Left/right	222/227	22/11	0.08
Height (cm)	165.3 ± 8.5	164.6 ± 7.2	0.68
Body weight (kg)	62.6 ± 11.6	61.1 ± 11.1	0.49
Surgical timing after injury (days)	78 (50–172)	69 (41–162)	0.26
General joint laxity	1 (0–2)	1 (0.5–3.5)	0.14
Hyperextension knee	48 (12.1%)	7 (24.1%)	0.14
Tegner Activity Scale	6.5 ± 1.3	7.2 ± 0.8	<0.01
Pivoting sports	226 (50.3%)	30 (90.9%)	<0.01
MM injury	148 (33.0%)	10 (30.3%)	0.85
LM injury	138 (30.7%)	9 (27.3%)	0.85
Posterior tibial slope angle	9.6 ± 2.9	11.9 ± 2.3	<0.01

*Note*: Data are expressed as means ± standard deviations, medians (interquartile ranges) and *n* (%). General joint laxity was scored using the University of Tokyo test (scores: 0–7) [21].

Abbreviations: LM, lateral meniscus; MM, medial meniscus.

### Surgical technique

In most cases, the modified transtibial technique was used for femoral tunnel creation [27]. However, in cases in which anatomical positioning of the tunnel was deemed difficult, an outside‐in or transportal technique was employed, with multiple primary surgeons performing the surgeries. Graft fixation involved the use of a suspensory device on the femoral side, with the choice of a button or post screw on the tibial side, depending on the surgeon's preference. Using a Tensioning Device (Arthrex), the graft was secured in a fully extended knee position with a tension ranging between 60 and 80 N. The graft was fixed at a tension considered optimal for the patient and evaluated intraoperatively using the pivot‐shift test [11].

### Post‐operative rehabilitation

A uniform post‐operative protocol was implemented for all patients [11]. Jogging was permitted at 2 months, followed by running at 4 months and non‐contact practice at 5 months, contingent upon achieving muscle strength levels of at least 75% compared to the healthy side. After 8 months, patients were cleared for participation in sports if their muscle strength reached a minimum of 80% of that of the healthy side. Subsequently, upon surpassing the 90% muscle strength threshold, the patients were individually assessed for their overall practical performance to determine their readiness for sports. These ‘return‐to‐sports’ criteria were based on the definitions published by Ardern et al. [3].

### Post‐operative graft failure

Graft failure was defined as obvious traumatic re‐rupture, graft rupture observed upon follow‐up MRI without a post‐operative trauma episode, and a side‐to‐side difference of ≥5 mm revealed by an arthrometer (Kneelax3; Monitored Rehab Systems). MRI was performed 8 months and 2 years post‐operatively to evaluate graft maturation. Additionally, arthrometer evaluations were conducted 5 months, 8 months, 1 year and 2 years post‐operatively. Arthrometry was performed with the difference between the surgical and healthy sides measured at 132 N and the knee flexed at 30° [11].

### Evaluation of GJL

GJL was evaluated using the University of Tokyo test following the established protocol [21]. The criteria for determining positive joint laxity included: (1) passive thumb opposition to the flexor aspect of the forearm for the wrist, (2) elbow hyperextension beyond 15°, (3) finger overlapping or grasping of the shoulder, (4) knee hyperextension of >10°, (5) ankle dorsiflexion of 45°, (6) trunk flexion with extended knees and both palms touching the mat for the spine and (7) toes pointing outwards beyond 180° for the hip. Each criterion was assigned one point, resulting in a total score of 7 points. Patients who showed a total score of 3 or more points were defined as being GJL positive.

### Evaluation of PTS

**Figure 2:**
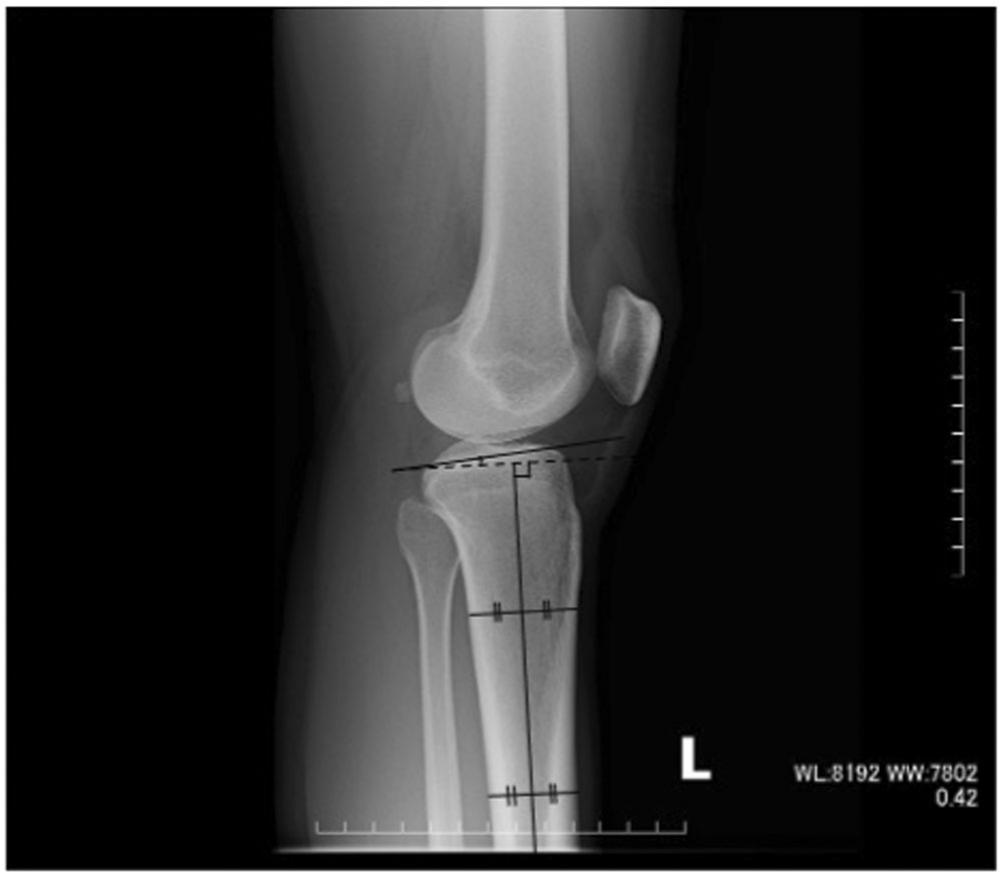
Lateral radiograph of the knee in the sagittal plane. Measurement of the posterior tibial slope angle: The posterior tibial slope angle was determined as the angle between the line perpendicular to the tibial mechanical axis and the line connecting the most proximal anterior and posterior points of the medial tibial plateau [25].

Pre‐operative lateral radiographic short films obtained with the knee maximally extended were used to evaluate the PTS angle [25]. Since previous studies have shown that the MTP is more commonly measured than the LTP on plain X‐rays [20], we adopted the measurement angle of the MTP in this study (Figure 2). The first author, who has over 10 years of experience in orthopaedic surgery, carefully measured each case in the entire cohort. To evaluate the reliability of these measurements, both intra‐observer and inter‐observer reproducibility were assessed using intraclass correlation coefficients (ICCs), which are suitable for evaluating consistency in repeated measurements of the same type. The first author performed the measurements three times for each knee, while two experienced surgeons each measured 10 randomly chosen pre‐operative knees once. The intra‐observer ICC was 0.87, indicating excellent consistency in the first author's repeated measurements. Moreover, the inter‐observer ICC was 0.72, indicating good agreement among measurements made by different surgeons.

### Statistical analyses

All statistical analyses were performed using R software (version 4.2.1; R Development Core Team). Mean values and standard deviations were used for continuous variables that showed a normal distribution, whereas medians and interquartile ranges were used for continuous variables that did not show a normal distribution. We used Student's *t* tests, Mann–Whitney *U* tests and chi‐square tests to compare means, medians, and nominal variables, respectively, when comparing clinical outcomes, demographic variables, pre‐operative variables and intraoperative variables between the two groups.

Moreover, receiver operating characteristic (ROC) curve analysis was used to calculate the cut‐off values for PTS and age, and the determined cut‐off values were used to dichotomise the variables. Logistic regression analysis was performed to identify significant risk factors for post‐operative graft failure. Variables with significant differences in the univariate analysis were used as independent variables, with post‐operative graft failure serving as the dependent variable. To identify risk factors for post‐operative graft failure, we calculated ORs and 95% confidence intervals (CIs) for all independent variables. Additionally, a stratified analysis was performed to determine the graft failure rate in patients with the identified risk factors.

In this study, we used one‐tailed hypothesis tests for known risk factors such as age and PTS, as previous studies have indicated the directionality of their effects [8, 21, 23, 32, 33]. Additionally, G‐power 3.1 software (Kiel University) was used to conduct post‐hoc power analysis for logistic regression, using an *α* of 0.05. The power analysis was based on a sample size of 482 knees, an observed graft failure rate of 6.8%, and ORs of 10.1 for age, 5.6 for PTS and 6.0 for pre‐operative participation in pivoting sports. These values were derived from the logistic regression results. This indicated that the powers for age, PTS and pre‐operative participation in pivoting sports were 0.95, 0.95 and 0.95, respectively. Based on these inputs, the effect sizes were calculated using G‐power's standard logistic regression formula. Additionally, we calculated Pearson's correlation coefficient to examine the independence of age and PTS as risk factors for graft failure.

## RESULTS

**Figure 3:**
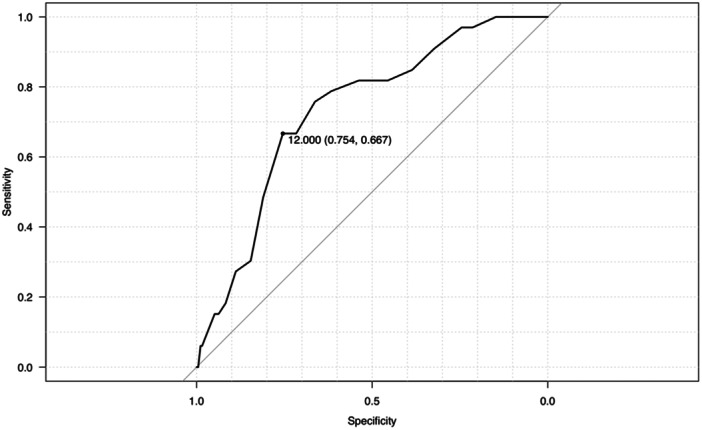
Receiver operating characteristics curve analysis for the posterior tibial slope angle. The area under the receiver operating characteristic curve was measured to be 0.734, with a cut‐off value of 12.0°, sensitivity of 0.667 and specificity of 0.754.

**Figure 4:**
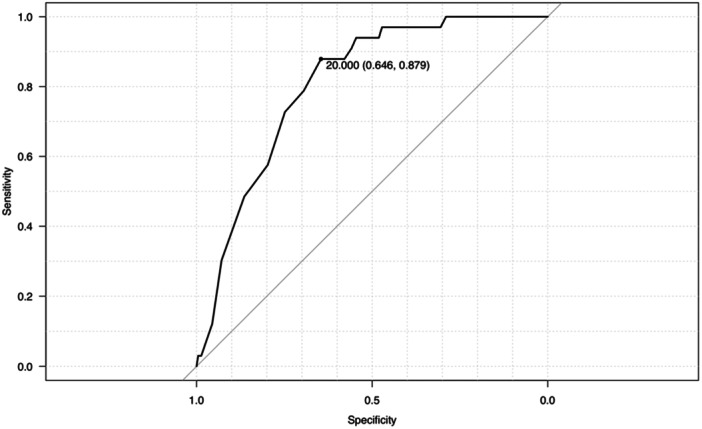
Receiver operating characteristics curve analysis for the patients' age (years). The area under the receiver operating characteristic curve was measured to be 0.808, with a cut‐off value of 20.0 years, sensitivity of 0.879 and specificity of 0.646.

In total, graft failure was observed in 33 out of 482 knees (6.7%). Of the 33 knees with graft failure, 31 resulted from re‐rupture and 2 were classified as failures based on a side‐to‐side difference of ≥5 mm in the Kneelax measurements. In the graft failure group, the proportion of pre‐operative pivoting sports was significantly higher, the pre‐operative Tegner Activity Scale was higher, the tibial slope was steeper and the patients were younger (Table 1). However, there were no significant differences in sex, meniscal injury, GJL or tibial hyperextension between the groups (Table 1). The cut‐off angle for PTS was found to be 12.0°, with a specificity of 70.9%, sensitivity of 69.7%, and area under the curve (AUC) of 0.734 (Figure 3). Moreover, the cut‐off value for age was 20.0 years, with a specificity of 64.6%, a sensitivity of 87.9% and an AUC of 0.808 (Figure 4).

**Figure 5:**
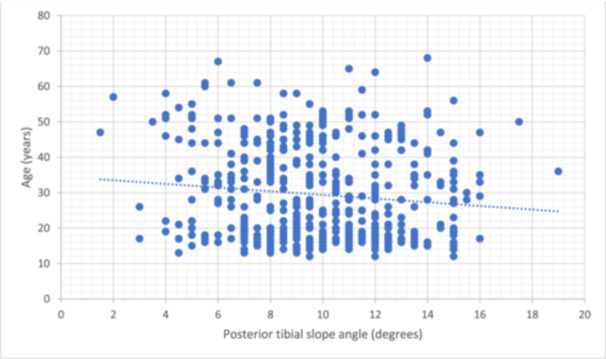
Analysis demonstrating the correlation between age and the posterior tibial slope angle.

Consequently, logistic regression analysis was performed using pre‐operative pivoting sports, the pre‐operative Tegner Activity Scale, an age of <20 years, and a PTS of ≥12° as explanatory variables. The ORs for pre‐operative pivoting sports, an age of <20 years, and a PTS of ≥12° were 6.01 (95% CI = 1.65–21.9; *p* < 0.01), 10.1 (95% CI = 3.34–30.5; *p* < 0.01) and 5.63 (95% CI = 2.46–12.9; *p* < 0.01), respectively (Table 2). The graft failure rate in patients with these three risk factors was 46.3% (Table 3). Notably, there was no correlation between age and tibial slope (correlation coefficient = −0.011; *p* = 0.012) (Figure 5).

**Table 2:** The results of logistic regression analysis.

Parameter	*p*	Odds ratio	95% Confidence interval
Age: <20 years	<0.01	10.10	3.34–30.50
PTS: >12°	<0.01	5.63	2.46–12.90
Pivoting sports	<0.01	6.01	1.65–21.90
Tegner Activity Scale	0.28	1.30	0.80–2.00

Abbreviation: PTS, posterior tibial slope.

**Table 3:** The graft failure rate of each risk factor according to stratified analysis.

Risk factor	Total	Graft failure	Graft failure rate (%)
None	116	0	0
Age <20 years or PTS > 12° or pivoting sports	231	5	2.2
Age <20 years + PTS > 12° or pivoting sports	94	9	9.6
Age <20 years + PTS > 12° + pivoting sports	41	19	46.3

Abbreviation: PTS, posterior tibial slope.

## DISCUSSION

The most important finding in this study was that age of <20 years, PTS of ≥12° and participation in pivoting sports were risk factors for graft failure after DB‐ACLR. Notably, 46.3% of the patients with these three risk factors experienced graft rupture. In contrast, none of the 116 patients without any of these risk factors experienced graft failure.

Many previous studies have investigated the risk factors for revision or re‐rupture after ACLR [29, 38]. Consensus among national registry big data, sophisticated meta‐analyses, and systematic reviews has indicated that younger age and steeper PTS, as well as the use of hamstring grafts instead of bone‐patellar tendon‐bone (BTB) grafts and allografts instead of autografts, are significant risk factors for revision or re‐rupture after ACLR [10, 18, 26, 38]. Conversely, studies have presented controversial results regarding the effect of factors such as sex, meniscal injury, and waiting time for surgery on graft failure [9, 29, 38]. For example, some studies have reported higher failure rates in patients with shorter waiting times; however, these patients often included a higher proportion of younger individuals, suggesting a significant age effect [29]. In contrast, other studies have suggested that longer waiting times can lead to postoperative instability [1]. This is based on the theory that longer waiting times increase the rate of meniscal injury [30] and the progression of anterior tibial subluxation [36]. In the present study, we observed no significant differences in waiting times for surgery; however, rotational laxity was not investigated, allowing the possibility that waiting times might affect rotational laxity, as suggested in previous studies [16, 31]. Although we observed significant differences in the types and levels of sports in the univariate analysis, consistent with previous studies, logistic regression analysis did not identify these as risk factors. This may be owing to a higher number of younger patients participating in pivotal and high‐level sports.

Regarding PTS, there is currently no consensus on the optimal measurement methods or thresholds [20]. Given the variability in MRI conditions and coverage among facilities, achieving highly reproducible and accurate measurements is challenging, rendering X‐ray measurements more reliable [13]. Moreover, in the cases examined at our institution, many MRI images did not sufficiently extend distally to show the tibial bone axis. Therefore, in the present study, we prioritised the versatility of the examination and adopted the measurement values from X‐ray examinations. Although controversy remains regarding whether the medial PTS (MPTS) or lateral PTS (LPTS) should be adopted when measuring the PTS, a systematic review by Liu et al. [20] indicated that most studies using X‐rays adopted the MPTS. Furthermore, another study that measured both the MPTS and LPTS and conducted ROC analysis on the risk of ACL rupture found that the MPTS had a higher sensitivity, specificity, and AUC than the LPTS [15]. Therefore, the MPTS was adopted in this study.

Few previous studies have determined the thresholds for graft failure using ROC curves [15, 23], and many have set values arbitrarily [5, 14, 17]. In contrast, in the present study, we calculated the ROC curves for graft failure and investigated its correlation with age and PTS. The novelty of our study lies in these investigations. We found that PTS is an independent risk factor, with a threshold of 12°. Notably, the MPTS values (cut‐off value of 12° and median value of 9.6° in cases without graft failure) measured using radiography in this study are similar to those of the current consensus [6, 20].

In the present study, the graft failure rate in patients with all three risk factors was as high as 46.3%, whereas that at 2 years post‐operatively was 0% in patients without any risk factors. Although a direct comparison with other grafts was not conducted, the 2‐year graft failure rate for patients with zero or one risk factor was 0%–2.2%. This suggests that DB‐ACLR may be a good option for patients with one or fewer of the risk factors identified in this study.

This study had several limitations. First, its retrospective nature introduces an inherent selection bias. The cut‐off values derived from ROC analysis are specific to this cohort, and clinical decision‐making should consider additional factors beyond these thresholds. Second, the high rate of loss to follow‐up (over 50%) may have introduced bias and affected the generalizability of the results. While no significant differences in baseline characteristics were found between those who completed follow‐up and those who lost to follow‐up, this limitation could still impact the overall conclusions. Third, while the inclusion of multiple surgeons led to non‐uniform surgical techniques, this variability could also be considered a strength of the study. It enhances the generalizability of findings by reflecting real‐world clinical practice, where surgical techniques often vary between surgeons. Fourth, tunnel positioning was not evaluated. Therefore, non‐anatomical tunnel placement may be a potential cause of failure. Fifth, the meniscal injuries were not classified according to location or type, which may have influenced our results. Injuries to the posterior horn and ramp lesions significantly affect post‐operative stability [4, 12], suggesting that a more detailed classification could have identified specific characteristics in the failure group. The Nagelkerke *R*² was calculated to assess the model fit in this study; this had a value of 0.33, indicating moderate explanatory power. However, this also suggests that other unmeasured factors likely contributed to graft failure. Therefore, future studies should aim to improve model robustness using cross‐validation and considering additional variables. Finally, alternative graft types or techniques were not compared, and overfitting remains a potential issue. Future research should investigate these aspects to strengthen the findings.

Despite these limitations, the risk factors detected in this study align with those in previous research [5, 8, 17, 21, 23, 33, 38], and this detailed investigation of 2‐year outcomes in a large cohort provides important insights into the risk factors for graft failure. We revealed that PTS and age are independent risk factors for graft failure, with ROC analysis providing an evidence‐based threshold. Therefore, the clinical significance of this study lies in the provision of evidence‐based information on the indications for DB‐ACLR. Specifically, we found that DB‐ACLR may be a good option for patients who are aged over 20 years, do not participate in pivoting sports, and have a tibial slope of less than 12°.

## CONCLUSION

An age of <20 years, PTS of ≥12° and participation in pivoting sports are risk factors for graft failure in DB‐ACLR. Approximately half of the patients with all three risk factors experienced graft failure, whereas the graft failure rate was 0% in patients without any risk factors.

## AUTHOR CONTRIBUTIONS

Kazumi Goto and Takaki Sanada contributed to the study conception. Takaki Sanada developed the theory and performed the computations. Kazumi Goto and Takaki Sanada verified the analytical methods. Takaki Sanada encouraged Kazumi Goto to investigate this study and supervised the findings of this work. Kazumi Goto designed the study and wrote the initial draft of the manuscript. Takaki Sanada contributed to the analysis and interpretation of data and assisted in manuscript preparation. All other authors contributed to data collection and interpretation and critical review of the manuscript. Takaki Sanada was in charge of overall direction and planning. All authors approved the final version of the manuscript and agreed to be accountable for all aspects of the work in ensuring that questions related to the accuracy or integrity of any part of the work are appropriately investigated and resolved.

## CONFLICT OF INTEREST STATEMENT

The authors declare no conflict of interest.

## ETHICS STATEMENT

This retrospective case‐control study was approved by the institutional review board of Kanto Rosai Hospital (2024‐12). Written informed consent was obtained from patients for participation in this case series and for publication of this case series and accompanying images.

## Data Availability

The data sets collected and/or analysed during the current study are available from the corresponding author upon reasonable request.
